# Exploring the Experiences of Health Visitors and Family Nurse Practitioners to Inform Digital Service Delivery

**DOI:** 10.1192/bjo.2026.11387

**Published:** 2026-06-30

**Authors:** Manuel Giardino, Nathan Hodson, Peter Woods, Rosie Gorringe, Juan Luque Solano

**Affiliations:** 1 Warwick Medical School, Coventry, United Kingdom; 2 Coventry and Warwickshire Partnership NHS Trust, Coventry, United Kingdom; 3 University of Buckingham Medical School, Milton Keynes, United Kingdom

## Abstract

**Aims:**

The early years of a child's life, particularly from birth to two years, are critical forcognitive, emotional, and social development. In the UK, Health Visitors and Family Nurse Practitioners (FNPs) play a pivotal role in supporting families during this period by delivering the Healthy Child Programme, offering guidance, and implementing early interventions to reduce health inequalities. These practitioners face significant challenges, including high caseloads, workforce shortages, and limited resources, which impact both the quality and quantity of care. This study explores the experiences of Health Visitors and FNPs, examining systemic barriers and identifying opportunities for innovation. It also evaluates the potential of digital tools, like the Pause App, to address gaps, foster empathetic and effective support, and improve outcomes for families and practitioners. By amplifying practitioners' voices through interviews, this study aims to inform policies and practices that better equip professionals to meet the diverse needs of parents and young children.

**Methods:**

Semi-structured interviews were conducted with 25 professionals, including Health Visitors and FNPs. Discussions explored their roles, programme advantages and disadvantages, challenges in practice, parent feedback, and available tools. Interviews were audio-recorded and analysed using Braun and Clarke’s thematic analysis approach. Coding was performed collaboratively using Taguette software. Themes were identified, refined, and validated through group discussions, with representative quotes selected to illustrate findings.

**Results:**

The analysis revealed key themes. Several challenges emerged as dominant themes: individual struggles, service constraints, and balancing competing responsibilities. Current tools were discussed extensively, with participants highlighting gaps, and areas for improvement. Engagement with families was identified as a critical factor in achieving positive outcomes, particularly in vulnerable populations. Trust was identified as a multifaceted theme, addressing relationships with parents, social services, and the extendedfamily context. Parent characteristics such as vulnerability, language and cultural differences, and balancing work, school and parenting were all factors that influenced service delivery. Feedback from practitioners underscored the importance of addressing needs such as mental health and safeguarding. These findings highlight the complexity of supporting families and the necessity of addressing systemic, cultural, and individual factors to enhance service effectiveness.

**Conclusion:**

This study highlights the challenges faced by practitioners in supporting families during early childhood. Despite systemic barriers like workforce shortages and limited resources, innovative tools such as the Pause App offer opportunities to improve services. Addressing gaps, fostering trust, and prioritising tailored support can enhance the effectiveness and sustainability of early years interventions.

